# Supplementary material for: High-Throughput UHPLC-MS to Screen Metabolites in Feces for Gut Metabolic Health
Source: Metabolites. 2022 Feb 25;12(3):211. doi: 10.3390/metabo12030211 (PMC8950041; doi:10.3390/metabo12030211)
Supplement: Supplementary file 1 [file metabolites-12-00211-s001.zip › metabolites-1542881-supplementary.pdf]

SUPPORTING INFORMATION

High-Throughput UHPLC-MS to Screen Metabolites in Feces for Gut Metabolic Health

Andressa de Zawadzki [1], Maja Thiele [2,3], Tommi Suvitaival [1], Asger Wretlind [1], Min Kim [1,4], Mina Ali [1], Annette F. Bjerre [1], Karin Stahr [1], Ismo Mattila [1], Torben Hansen [5], Aleksander Krag [2,3] and Cristina Legido-Quigley [1,6,*]

1 Steno Diabetes Center Copenhagen, 2730 Herlev, Denmark; andressa.de.zawadzki@regionh.dk (A.d.Z.); tommi.raimo.leo.suvitaival@regionh.dk (T.S.); asger.wretlind@regionh.dk (A.W.); min.kim@dbac.dk (M.K.);
mina.ali@regionh.dk (M.A.); annette.frost.bjerre@regionh.dk (A.F.B.); karin.stahr.01@regionh.dk (K.S.); ismo.matias.mattila@regionh.dk (I.M.)

2 Department of Gastroenterology and Hepatology, Odense University Hospital, 5000 Odense, Denmark; maja.thiele@rsyd.dk (M.T.); aleksander.krag@rsyd.dk (A.K.)

3 Department of Clinical Medicine, University of Southern Denmark, 5230 Odense, Denmark

4 Copenhagen Prospective Studies on Asthma in Childhood, Herlev and Gentofte Hospital, 2730 Herlev, Denmark

5 Novo Nordisk Foundation Center for Basic Metabolic Research, Faculty of Health and Medical Sciences, University of Copenhagen, 1165 Copenhagen, Denmark; torben.hansen@sund.ku.dk

6 Institute of Pharmaceutical Science, King's College London, London SE19NH, UK

* Correspondence: cristina.legido.quigley@regionh.dk

Abstract: Feces are the product of our diets and have been linked to diseases of the gut, including Chron's disease and metabolic diseases such as diabetes. For screening metabolites in heterogeneous samples such as feces, it is necessary to use fast and reproducible analytical methods that maximize metabolite detection. As sample preparation is crucial to obtain high quality data in MS-based clinical metabolomics, we developed a novel, efficient and robust method for preparing fecal samples for analysis with a focus in reducing aliquoting and detecting both polar and non-polar metabolites. Fecal samples (n = 475) from patients with alcohol-related liver disease and healthy controls were prepared according to the proposed method and analyzed in an UHPLC-QQQ targeted platform in order to obtain a quantitative profile of compounds that impact liver-gut axis metabolism. MS analyses of the prepared fecal samples have shown reproducibility and coverage of n = 28 metabolites, mostly comprising bile acids and amino acids. We report metabolite-wise relative standard deviation (RSD) in quality control samples, inter-day repeatability, LOD, LOQ, range of linearity and method recovery. The average concentrations for 135 healthy participants are reported here for clinical applications. Our high-throughput method provides a novel tool for investigating gut-liver axis metabolism in liver-related diseases using a noninvasive collected sample.

Keywords: bile acids; fecal metabolomics; gut-liver axis; targeted metabolomics; sample preparation

Supporting information

Table of contents

Table S1. MS parameters of the dynamic MRM method that was optimized from Ahonen et al., 2019 for detection of metabolites in fecal samples. Parameters include MRM transition, polarity, retention time, fragmentor voltage (V), collision energy (V). **3**

Table S2. MS parameters of the dynamic MRM method that was optimized from Ahonen et al., 2019 for detection of the internal standards in fecal samples. Parameters include MRM transition, polarity, retention time, fragmentor voltage (V), collision energy (V). Final concentration of internal standards (IS) in the ISTDmix in ng mL^{-1} and was determined for each compound based on their limits of quantification. **5**

Table S3. Metabolites identified by GCGC-MS in fecal samples from healthy controls. Columns represent the classes of compounds that belong to fatty acid metabolism, amino acid metabolism, carbohydrate metabolites, TCA and others. **6**

Table S4. Limit of detection (LOD); limit of quantification (LOQ); linearity (R2); linear regression parameters for calibration curves: (A) slope and (B) intercept with standard deviation (SD); range of linearity by UHPLC-MS analysis of bile acids and amino acids. **7**

Table S5. Metabolite-wise relative standard deviation (RSD) in pooled samples (PO), study samples ("sample"), estimated ratio between biological variation and technical variation ("ratio"), inter-day repeatability for two different concentrations (conc. 1 and conc. 2) in the range of LLOQ and ULOQ and, relative recovery calculated for samples spiked with standards at low (LS), medium (MS) and high concentrations (HS). **8**

Figure S1. Study design for evaluation of method reproducibility in a UHPLC-MS targeted platform. Quality control (QC) samples including blanks, calibration solutions and pooled samples were distributed throughout 600 injections. Four calibration curves were placed at the beginning, middle and at the end of the analysis. Analysis started with 2 blanks, 3 pooled samples, a calibration curve with 11 levels of concentration and 1 blank. A unit composed by a blank, a pool and 16 clinical samples was repeated until the next calibration curve. **9**

Figure S2. Analytical stability of a targeted UHPLC-MS platform for quantitative analysis of metabolites related to gut-liver axis. Time related variation of metabolite response measured in quality control pooled samples representing replicates and samples representing a cohort. Interval of variation in QC pooled samples is represented in grey. **10**

Figure S3. Violin plots for distribution of metabolite concentrations across human fecal samples representing a cohort with different degrees of liver disease (n=475) and fecal pooled samples (PO), used as replicates (n=36) for quality control. **11**

Table S1. MS parameters of the dynamic MRM method that was optimized from Ahonen et al., 2019 for detection of metabolites in fecal samples. Parameters include MRM transition, polarity, retention time, fragmentor voltage (V), collision energy (V).

Compound	Abbreviation	Molecular Weight (MW)	Retention time	MRM transition	Polarity	Fragmentor Voltage (V)	Collision Energy (V)	Cell Accelerator Voltage (V)
DL-2-Aminoadipic Acid	AADA	161.2	2.6	330.2–160.1	Negative	150	10	1
Asymmetric dimethylarginine	ADMA and SDMA	202.3	2.4	371.2–201.2 *	Negative	150	5	5
			2.4	371.2–156.1	Negative	150	20	1
L-Alanine	Ala	89.1	2.6	258.1–88.1	Negative	100	15	3
Azelaic acid	AzelA	188.2	3.8	187.2–169	Negative	150	10	2
			3.8	187.2–125.2 *	Negative	150	15	2
L-3-hydroxybutyric Acid	β-OHB	104.1	0.7	103.2–59.2	Negative	100	5	1
Cholic Acid	CA	408.6	6.4	407.3–407.3 *	Negative	250	10	3
			6.4	407.3–343.3	Negative	250	35	1
Deoxychenocholic Acid	CDCA	392.6	7.0	391.3–391.3	Negative	200	10	4
L-Citrulline	Cit	175.2	2.5	344.4–174.2	Negative	150	4	7
Deoxycholic Acid	DCA	392.6	7.0	391.2–345.3 *	Negative	200	35	4
			7.0	391.2–327.2	Negative	200	40	4
Gamma-butyrobetaine	GBB	146.2	0.85	147.2–88.1 *	Positive	100	16	1
			0.85	147.2–60.2	Positive	100	13	1
Glycocholic Acid	GCA	465.6	5.95	464.3–402.1	Negative	250	40	4
			5.95	464.3–74.1 *	Negative	250	45	7
Glycochenodeoxycholic Acid	GCDCA	449.6	6.5	448.3–448.3	Negative	200	40	2
	GCDCA	449.6	6.5	448.3–74.1	Negative	200	55	4
Glycodeoxycholic Acid	GDCA	449.6	6.6	448.3–74.2	Negative	200	55	4
Glycolithocholic acid	GLCA	433.3	7.1	432.3–388.1	Negative	200	25	4
				432.3–74.1*	Negative	200	45	4
L-Glutamine	Gln	146.1	2.4	315.3–145.1	Negative	100	9	6
L-Glutamic Acid	Glu	147.1	2.45	316.1–146.1	Negative	100	6	6
Glycine	Gly	75.1	2.4	244.1–74.1	Negative	200	7	4
Glycoursodeoxycholic Acid	GUDCA	449.6	6.0	448.3–386	Negative	250	40	2
			6.0	448.3–74.1 *	Negative	250	45	2
L-Homocitrulline	HCit	189.2	2.6	358.3–188.1	Negative	200	10	1
			2.6	358.3–145 *	Negative	150	25	2
Indoxyl Sulfate	IndS	213.2	2.55	212–132 *	Negative	100	15	2
			2.55	212–80	Negative	100	20	2
L-Kynurenine	Kynu	208.2	3.65	377–316.1	Negative	150	5	2

Compound	Abbreviation	Molecular Weight (MW)	Retention time	MRM transition	Polarity	Fragmentor Voltage (V)	Collision Energy (V)	Cell Accelerator Voltage (V)
			3.65	377–207 *	Negative	150	5	5
Lithocholic acid	LCA	376.3	7.6	375.3–375.3	Negative	240	10	4
			7.6	375.3–45	Negative	240	100	4
L-Leucine	Leu and Ile	131.2	3.72	300.2–130.2	Negative	100	10	1
L-Isoleucine	Ile	131.2	3.6	300.2–130.2	Negative	100	10	1
N-methyl-nicotinamide	N-MNA	136.2	1.05	137.1–108.1	Positive	100	15	2
				137.1–80.2 *	Positive	100	26	2
L-Phenylalanine	Phe	165.2	3.95	334.2–164	Negative	100	10	1
Taurine	Taurine	125.2	2.32	294.1–124.1 *	Negative	100	10	2
				294.1–80.1	Negative	100	55	2
Taurocholic Acid	TCA	515.7	5.7	514.3–123.8	Negative	300	65	5
			5.7	514.3–80.2 *	Negative	300	95	1
Taurochenodeoxycholic Acid	TDCA and TCDCA	499.3	6.05	498.3–107.1	Negative	250	80	4
			6.15	498.3–80.1 *	Negative	300	90	4
Taurodeoxycholic Acid		499.3	6.15	498.3–498.3	Negative	250	10	4
L-Tryptophan	Trp	204.2	4.27	373.2–203.1	Negative	150	7	2
Tauroursodeoxycholic Acid	TUDCA	499.7	5.7	498.3–107.1	Negative	250	65	5
				498.3–80.1 *	Negative	250	70	1
L-Tyrosine	Tyr	181.2	3.97	350.2–180.1	Negative	100	7	5
Ursodeoxycholic acid	UDCA	392.3		391.3–391.3	Negative	250	10	4
			7.0	391.3–289.3	Negative	250	45	4

*ion transition used for quantification;
MRM transitions of amino acids represent the ion transition for the adduct with AQC reagent.

Table S2. MS parameters of the dynamic MRM method that was optimized from Ahonen et al., 2019 for detection of the internal standards in fecal samples. Parameters include MRM transition, polarity, retention time, fragmentor voltage (V), collision energy (V). Final concentration of internal standards (IS) in the ISTDmix in ng mL^{-1} and was determined for each compound based on their limits of quantification.

Compound	IS concentration (ng mL^{-1})	Molecular Weight (MW)	Retention time (min)	MRM transition	Polarity	Fragmentor Voltage (V)	Collision Energy (V)	Cell Accelerator Voltage (V)
AADA-d3	30000	164.2	2.6	333.2–145.2	Negative	100	20	2
ADMA-d7	10000	209.8	2.45	378–208.3	Negative	100	10	5
Ala-d4	5000	93.1	2.6	262.1–92.1	Negative	100	5	6
AzelA-d14	5000	202.3	3.7	201.2–137.2	Negative	150	10	2
β-OHB-d4	100000	108.1	0.75	107.1–59.1	Negative	100	5	1
CA-d4	500	412.3	6.37	411.3–411.3	Negative	250	10	3
CDCA-d4 DCA-d4	500	396.6	7.0	395.2–395.2	Negative	300	10	4
Cit-d4	1000	179.2	2.47	348.1–135.1	Negative	100	25	2
GBB-d9	5000	154.7	0.87	155.2–87.3	Positive	100	13	1
GCA-d4	250	469.6	5.97	468.3–74.1	Negative	250	45	1
GCDCA-d4 GUDCA-d4	30000	453.6	6.55	452.3–74.1	Negative	250	40	1
GUDCA-d4	10000	453.6	6.55	452.3–74.1	Negative	250	40	1
GDCA-d6	30000	455.7	6.6	454.3–408.2	Negative	250	55	4
GLCA-d4	1000	437.3	7.1	436.3–74.1	Negative	200	50	4
Gln-d5	60000	151.2	2.46	320.1–150.1	Negative	100	5	1
Glu-d5	10000	152.1	2.46	321.1–151.1	Negative	100	5	1
Gly-13C,d2	10000	78.1	2.4	247–77.1	Negative	100	5	7
HCit-2H4	10000	193.2	2.6	362.2–192.2	Negative	100	5	6
IndS-d4	10000	217.3	2.55	216–136.1	Negative	100	15	2
Kynu-13C6	30000	214.2	3.6	383.1–195.8	Negative	100	10	6
LCA-d4	1000	380.3	7.55	379.3–379.3	Negative	240	100	4
Leu-d10 Ile-d10	5000	141.2	3.6 3.55	310.1–140	Negative	125	10	2
N-MNA-d4	750	140.2	1.3	141.2–84.2	Positive	100	20	7
Phe-d5	1000	170.2	3.9	339.1–169.1	Negative	150	5	1
Taurine-d4	1000	129.2	2.32	298.3–128.2	Negative	100	10	3
TCA-d4	500	519.7	5.7	518.3–80	Negative	340	100	7
TCDCA-d9	500	508.3	6.1	507.4–80.1	Negative	300	95	4
Trp-d8	5000	212.3	4.25	381.2–211.2	Negative	100	10	5
TUDCA-d4	250	503.7	5.7	502.3–80.1	Negative	250	70	1
Tyr-d7	5000	188.2	2.97	357.1–187.2	Negative	100	10	1
UDCA-d4	250	396.6	7.0	395.3–395.3	Negative	250	0	4

MRM transitions of amino acids represent the ion transition for the adduct with AQC reagent.

Table S3. Metabolites identified by GCGC-MS in fecal samples from healthy controls. Columns represent the classes of compounds that belong to fatty acid metabolism, amino acid metabolism, carbohydrate metabolites, TCA and others.

Free fatty acids		Amino acids	Carbohydrates	TCA metabolites /others
Stearic acid	Nonanedioic acid	Methionine	Myo inositol	Citric acid
Oleic acid	Tetradecanoic acid	Serine	d-Ribose	Fumaric acid
Palmitic acid	1,4-Butanediol	Glycine	L-(-)-Fucose	Succinic acid
Palmitoleic acid	1-Octadecanol	Leucine	Sorbitol	Malic acid
1-Monopalmitin	1-Dodecanol	Phenylalanine	Maltose	Lactic acid
Linoleic acid	3,4-Dihydroxybutanoic acid	Alanine	d-Galactose	3-Indoleacetic acid
Tetradecanoic acid	2,4-Dihydroxybutanoic acid	Proline	D-Arabinose	3-Hydroxyphenylacetic acid
Butanoic acid	4-Hydroxybutanoic acid	Valine	Lactic acid	Deoxycholic acid (bile acid)
Hexanoic acid	Benzoic acid	a-alanine	D-(-)-Fructofuranose	
Pentanoic acid	Triethylene glycol	cadaverine	a -D-Glucopyranose	
Heptanoic acid	Diethylene glycol		D-(+)-Galactopyranose	
Octanoic acid	Glyceric acid		alpha-ketoglutaric acid	
Nonanoic acid	Glycerol			
Decanoic acid	Glycerol monostearate			
Heptadecanoic acid	5-Aminovaleric acid			
Pentadecanoic acid	3-Methyladipic acid			

Table S4. Limit of detection (LOD); limit of quantification (LOQ); linearity (R2); linear regression parameters for calibration curves: (A) slope and (B) intercept with standard deviation (SD); range of linearity by UHPLC-MS analysis of bile acids and amino acids.

Compound	LOD (ng mL^{-1})	LOQ (ng mL^{-1})	R^2	slope	SD of slope	intercept	SD of intercept	LLOQ	ULOQ
AADA	253.87	769.30	0.951	0.00221	$3.93\ 10^{-6}$	15.26658	0.170031	1000	50000
ADMA	311.35	943.49	0.983	0.000184	$5.11\ 10^{-7}$	-0.11094	0.017319	1000	50000
Ala	0.03	0.084	0.978	26.20151	0.082371	-3.63255	0.221374	2	50000
AZelA	139.47	422.64	0.91	0.000597	$2.12\ 10^{-6}$	-0.50651	0.025227	500	25000
β-OHB	177.23	537.06	0.993	$8.04\ 10^{-6}$	$1.34\ 10^{-8}$	6.2210^{-5}	0.000432	750	10000
CA	70.78	214.49	0.999	0.00257	$2.05\ 10^{-6}$	-1.00791	0.055114	10	100000
CDCA	0.69	2.09	0.998	0.004932	$2.12\ 10^{-6}$	0.022947	0.001031	10	50000
Cit	217.05	657.71	0.992	$3.66\ 10^{-5}$	$7.11\ 10^{-8}$	-0.05226	0.002409	750	50000
DCA	7.915	23.98	0.995	0.000124	$7.69\ 10^{-7}$	0.000553	0.000296	25	100000
GBB	153.43	464.92	0.992	0.000186	$3.22\ 10^{-7}$	0.024853	0.008633	500	50000
GCA	12.36	37.44	0.999	0.001841	$2.20\ 10^{-7}$	-0.19432	0.006893	50	100000
GCDCA	3.81	11.54	0.969	0.015198	$3.29\ 10^{-5}$	0.056013	0.017533	50	50000
GDCA	12.71	38.51	0.989	0.009236	$8.67\ 10^{-5}$	-0.03423	0.035568	50	50000
GLCA	6.36	19.29	0.997	0.005836	$2.74\ 10^{-5}$	-0.00354	0.011255	50	100000
Gln	230.60	698.78	0.995	0.001883	$8.47\ 10^{-6}$	1.57648	0.131603	750	25000
Glu	174.43	528.58	0.969	0.000264	$4.18\ 10^{-7}$	0.162696	0.01398	500	50000
Gly	265.13	803.42	0.979	0.001627	$4.96\ 10^{-6}$	-2.15256	0.130738	1000	100000
GUDCA	5.90	17.88	0.999	0.000256	$1.28\ 10^{-8}$	-0.05641	0.000458	50	100000
HCit	295.98	896.90	0.985	0.000598	$1.58\ 10^{-6}$	-0.88806	0.053648	1000	50000
Ile	32.20	97.57	0.944	0.0016	$3.27\ 10^{-5}$	-0.01189	0.015609	100	50000
IndS	145.05	439.54	0.996	$3.09\ 10^{-5}$	$4.30\ 10^{-8}$	-0.02029	0.001358	500	100000
Kynu	171.43	519.47	0.972	0.481562	$7.42\ 10^{-4}$	-1045.78	25.0159	500	10000
LCA	7.09	21.49	0.998	0.00247	$1.11\ 10^{-5}$	0.016653	0.005307	10	100000
Leu	0.02	0.05	0.972	45.95384	0.16	-3.75385	0.250482	2	100000
N-MNA	11.29	34.23	0.994	0.000535	$4.23\ 10^{-6}$	-0.00196	0.001832	100	50000
Phe	34.75	105.31	0.941	0.00034	$7.50\ 10^{-6}$	-0.0028	0.003578	250	50000
Taurine	81.68	247.51	0.995	0.000618	$3.44\ 10^{-7}$	-1.45427	0.015284	250	50000
TCA	37.39	113.32	0.999	0.004373	$1.16\ 10^{-6}$	0.615186	0.049558	100	100000
TCDCA	0.07	0.21	0.998	0.033012	$4.10\ 10^{-6}$	-0.02961	0.000682	50	100000
TDCA	23.52	71.27	0.966	0.026576	0.000437	-0.12875	0.189402	100	100000
Trp	225.02	681.88	0.932	0.00255	$9.95\ 10^{-6}$	-6.27553	0.173872	750	100000
Tyr	165.60	501.82	0.992	0.000319	$4.92\ 10^{-7}$	-0.34754	0.016009	500	100000
UDCA	5.65	17.12	0.997	0.004278	$1.90\ 10^{-5}$	0.008358	0.007324	50	50000
TUDCA	60.80	184.23	0.997	0.003023	$1.30\ 10^{-6}$	-0.31566	0.05569	500	50000

Table S5. Metabolite-wise relative standard deviation (RSD) in pooled samples (PO), study samples ("sample"), estimated ratio between biological variation and technical variation ("ratio"), inter-day repeatability for two different concentrations (conc. 1 and conc. 2) in the range of LLOQ and ULOQ and, relative recovery calculated for samples spiked with standards at low (LS), medium (MS) and high concentrations (HS).

Compound	PO	Sample	Ratio	Inter-day repeatability (n=4)		Relative Recovery (%)		
				conc. 1	conc. 2	LS	MS	HS
GBB	12.9	2.03 10^3	158	4.2	2.2	82.3	114.8	105.0
GCDCA	13	1.73 10^3	133	9.9	15.3	95.8	106.1	104.8
GDCA	29	2.05 10^3	70.8	15.2	7.7	91.6	98.3	98.2
Trp	7.68	491	64	5.9	3.8	87.2	81.0	95.2
Ile	12.2	411	33.5	12.9	8.5	82.8	89.8	117.8
TUDCA	21.3	708	33.2	16.8	3.55	88.7	103.98	92.12
AzelA	14.4	477	33.2	7.1	19.8	97.6	91.5	87.6
CA	12.2	362	29.7	3.8	2.1	93.4	94.7	101.1
Leu	16.9	481	28.4	2.8	11.5	87.3	93.6	96.8
GUDCA	13.4	314	23.5	3.7	1.6	97.0	103.4	111.5
Ala	16.1	230	14.2	17.6	6.5	89.2	90.3	102.2
TDCA	15.7	247	15.7	9.7	21.4	86.9	115.2	94.8
Phe	11	170	15.6	3.6	1.9	81.3	81.0	119.4
TCDCA	20.5	304	14.8	4.2	12.5	94.1	102.2	82.0
Glu	16.2	219	13.5	26.6	10.6	97.4	94.9	-
GCA	22.9	300	13.1	3.2	1.7	113.9	96.6	80.1
Cit	14.9	99.6	6.68	2.3	8.5	114.3	103.0	80.6
TCA	13.2	65.8	5.0	4.1	8.2	94.1	102.3	82.0
Taurine	12.2	322	26. 3	2.5	5.6	107.0	86.5	102.0
Tyr	6.24	25.5	4.09	4.4	5.1	92.1	106.0	92.15
LCA	15.9	105	6.65	3.2	5.6	113.0	99.0	91.0
CDCA	29.1	100	3.44	3.4	2.6	102.3	100.8	88.2
DCA	9.79	88	8.89	6.4	1.1	80.2	86.1	86.1
β-OHB	21.5	273	11.8	5.0	13.8	81.9	107.2	111.3
Gln	28.9	108	3.0	11.5	26.0	86.4	105.9	-
IndS	128	182	1.42	6.5	20.0	-	-	-
Kynu	23.4	31	1.33	6.2	3.7	80.3	99.1	118.0
AADA	80.8	410	5.08	8.8	18.3	-	-	-
UDCA	24.0	116	4.84	12.8	4.3	-	92.6	104.4
GLCA	18.5	23.9	1.29	2.1	10.5	103.3	108.0	100.8
Gly[a]	-	-	-	16.7	16.5	-	-	-
N-MNA	41.7	99.2	2.38	16.9	4.7	-	-	-
ADMA	34.6	43.1	1.24	2.5	5.2	-	-	-
HCit	86.4	104	1.21	6.4	4.9	106.2	102.1	89.0

[a] non-detected in 50% of samples

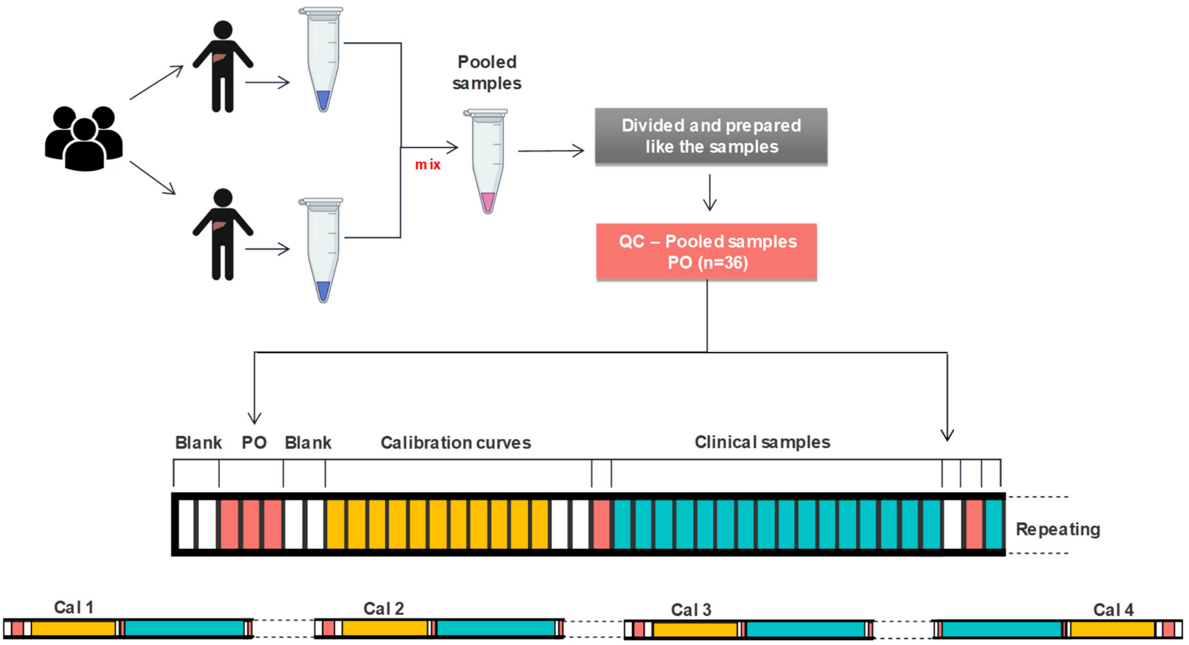

Figure S1. Study design for evaluation of method reproducibility in a UHPLC-MS targeted platform. Quality control (QC) samples including blanks, calibration solutions and pooled samples were distributed throughout 600 injections. Four calibration curves were placed at the beginning, middle and at the end of the analysis. Analysis started with 2 blanks, 3 pooled samples, a calibration curve with 11 levels of concentration and 1 blank. A unit composed by a blank, a pool and 16 clinical samples was repeated until the next calibration curve.

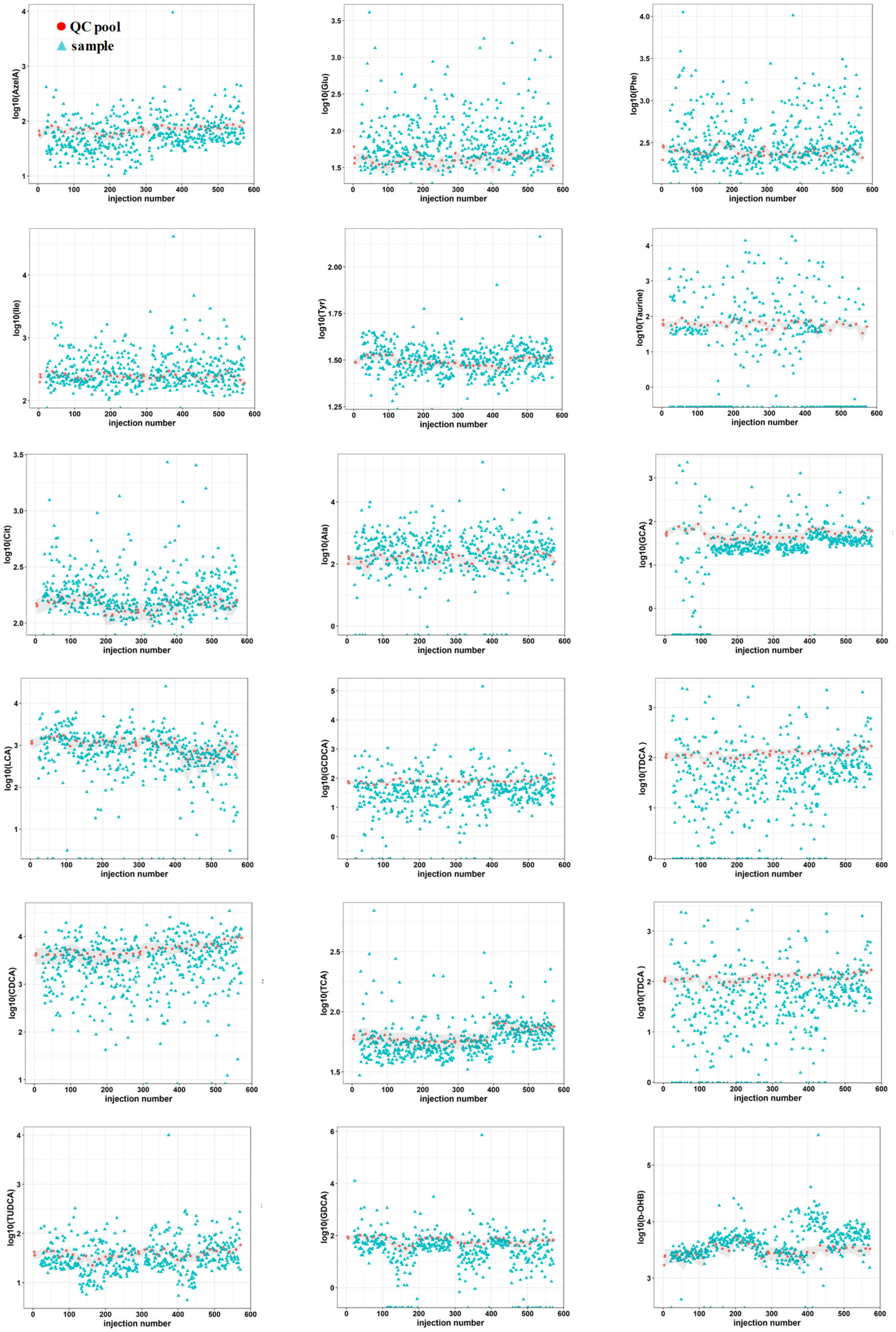

Figure S2. Analytical stability of a targeted UHPLC-MS platform for quantitative analysis of metabolites related to gut-liver axis. Time related variation of metabolite response measured in quality control pooled samples representing replicates and samples representing a cohort. Interval of variation in QC pooled samples is represented in grey.

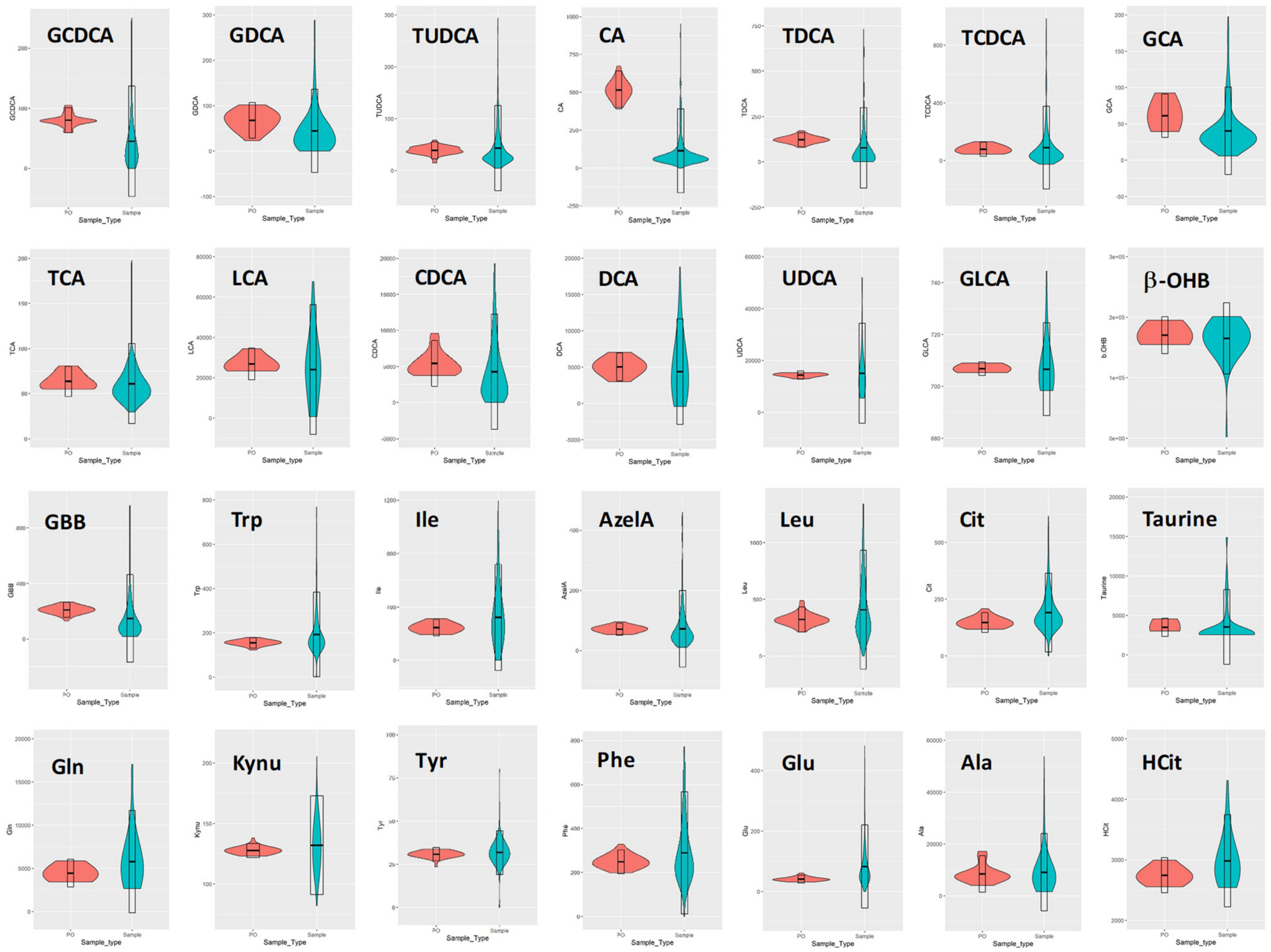

Figure S3. Violin plots for distribution of metabolite concentrations across human fecal samples representing a cohort with different degrees of liver disease (n=475) and fecal pooled samples (PO), used as replicates (n=36) for quality control.
